# Airway Management During Anesthetic Induction of Secondary Laryngectomy for Recurrent Laryngeal Cancer: Three Cases of Report and Analysis

**DOI:** 10.3389/fmed.2018.00264

**Published:** 2018-09-19

**Authors:** Xuezheng Zhang, Omer Cavus, Ying Zhou, Sasima Dusitkasem

**Affiliations:** ^1^Department of Anesthesiology, The First Affiliated Hospital of Wenzhou Medical University, Wenzhou, China; ^2^Department of Anesthesiology, Wexner Medical Center, The Ohio State University, Columbus, OH, United States; ^3^Department of Pulmonary Medicine, The First Affiliated Hospital of Wenzhou Medical University, Wenzhou, China; ^4^Department of Anesthesiology, Ramathibodi Hospital, Mahidol University, Bangkok, Thailand

**Keywords:** laryngeal cancer, recurrent, surgery, difficult airway, anesthesia

## Abstract

Surgery for laryngeal cancer and the following recurrent tumor growth may further change the anatomy of the airway. Airway management during anesthesia induction is challenging for the patients undergoing secondary surgery due to recurrence of laryngeal cancer or its postoperative complication, but it has never been reported. In this report, we described three cases of anesthetic induction which had different process of airway events. The first case was given intravenous general anesthetic for induction and experienced failed intubation, difficult mask ventilation and emergent tracheostomy, eventually were rescued successfully. The second case presented a fixed metastatic mass about 6 cm diameter upon the primary surgical scar of incision and preoperative apnea, underwent fibroscopy-guided conscious intubation and the process was uneventful. The third case had erythema and swelling under the mandible with erupted ulcer as well as neck immobility due to recurrent tumor. The anesthesiologist attempted fibroscopy-guided intubation via nasal passage with a tracheal tube in 2.8 mm diameter but it was failed. Subsequently, tracheostomy was performed under bilateral superficial cervical plexus block and the dissected larynx by operation verified distorted structure of glottis with S-shaped stenosis. This report concludes that, during the anesthetic induction for this special type of surgery, a detailed and comprehensive evaluation of the airway, and a routine fibroscopic examination are especially important.

## Background

Laryngeal cancer accounts for about 50% of head and neck malignant tumors and the ratio of male to female occurrence is 4:1 (1). The glottis (59%), the supraglottic area (40%), and the subglottic area (1%) are three major locations for laryngeal cancer (2). The main long-term complications after laryngeal cancer surgery are laryngeal stenosis, fistula formation, local recurrence of cancer, and adjacent and remote lymph node metastasis (3). However, for patients undergoing the secondary laryngeal cancer surgery, due to recurrence or postoperative complications, airway abnormalities may develop due to the previous laryngeal cancer surgery or the newly emergent malignancy. In addition, patency of the airway can be further complicated by laryngeal radiation therapy-induced fibrosis and reduced mobility of the surrounding tissues (4).

As for the anesthesiologist, airway related information may be acquired from medical history, physical examination, radiography, and from the surgeons. Furthermore, the techniques such as fibroscopy, jet ventilation, retrograde tracheal intubation, and tracheostomy as well as guidelines for difficult airway management should be well understood. All the associated necessary equipment should also be ready for an instant application (5). It has been well established that the presence of laryngeal cancer may lead to difficulties in airway management both in term of difficult mask ventilation and tracheal intubation (6, 7).

Based on the knowledge of the possible occurrence of difficult ventilation, additional measures such as preparation of emergent tracheostomy, cardiopulmonary resuscitation, and airway management following ASA guideline may be implemented. In this report, we want to draw the attention to the importance of detailed airway evaluation and fibroscopic examination before recurrent laryngeal cancer surgery.

## Case presentations

### Case one

A 54-years old male, weighing 66 kg, with tobacco use for 30 years, had undergone the primary partial laryngectomy 13 months before. He was scheduled for total laryngectomy and neck dissection. No abnormality was detected with preoperative physical examination and CT scan showed increased lung markings without metastasis. It also demonstrated moderate general condition with 3 cm of mouth opening, ECG with right bundle block, Malampatti grade II, without complaint of apnea or major depression signs. There was an 8 cm healed scar of a previous surgical incision along the cervical midline but with normal neck extension. Routine non-invasive blood pressure, ECG, and SPO2 were monitored. General anesthesia was induced with midazolam (1 mg), sufentanil (20μg), vecuronium (8 mg) and propofol (120 mg) followed by an attempted tracheal intubation using Macintosh laryngoscopy after preoxygenation. Nonetheless, the epiglottis and glottis were not viewed under Macintosh laryngoscopy, and then SPO2 dropped to 85% from 98%. Meanwhile, no effective ventilation was obtained by mask ventilation. The subsequent placement of neither an oropharyngeal airway nor a laryngeal mask airway (LMA) could resolve the difficult ventilation, which was accompanied by a quick drop of SPO2 to 25%, and an increased heart rate to 150 beats per minutes. In a short time, urgent tracheostomy was performed by a standby otolaryngologist, and then SPO2 and heart rate recovered after oxygen delivery through the placement of a coiled tracheal tube of 7.0 mm diameter. Anesthesia was maintained with continuous infusion of propofol, remifentanil, and inhalational sevoflurane. The patient emerged without sequelae from the short episode of hypoxia after the surgery was over.

### Case two

A 57-year old male, weighing 66 kg, who had undergone semi-laryngectomy one year ago, was scheduled for total laryngectomy. The ECG was normal, and his blood pressure was 130/80 mmHg. The patient with Malampatti grade II had slight inspirational apnea which would be exacerbated by head lift. Therefore supine position was maintained. The lowest SPO2 of the patient was 90% under room air. Preoperative physical examination revealed that there was a fixed metastatic mass about 6 cm diameter upon the primary surgical scar of the previous incision along the cervical midline. CT scan of the neck reported malignant invasion of the tracheal wall. To prevent the slight apnea exacerbation, fibroscopy-guided conscious intubation was planned. The fibroscopy-guided tracheal intubation was performed successfully after administration of intravenous midazolam (1 mg), fentanyl (50 μg), and glottic topical anesthesia with 2% lidocaine (4 ml) spray through the suction channel of the fibroscope. After that, we proceeded with general anesthesia immediately and the surgery was uneventful.

### Case three

A 63-years old male, diagnosed with recurrent laryngeal cancer after semi-laryngectomy 8 months ago, was scheduled to undergo total laryngectomy. He has 17 years' history of hypertension and his echocardiography revealed mild impairment of the left ventricular diastolic function. He has normal mouth opening and the airway was evaluated as Malampatti grade III. There were erythema and swelling under his right mandible with an erupted ulcer as well as neck immobility which were caused by tumor invasion. Non-invasive BP, ECG, and SPO2 were monitored. The preoperative blood pressure was 180/95 mmHg and was reduced to 130/80 mmHg following intravenous administration of urapidil (25 mg). After topical application of 2% lidocaine (4 ml) to the tracheal and oropharyngeal areas, intravenous injection of midazolam (1 mg) and fentanyl (50 μg) was followed by the fibroscope-guided nasal intubation. However, obvious structural deformation of the larynx was presented with impaired vocal cord mobility leaving only a“fissure” of space which thwarted the passage of the fibroscope even with 2.8 mm diameter. Subsequently, tracheostomy was performed under bilateral superficial cervical plexus block with assisted mask ventilation by 100% oxygen followed by 7.0 mm coiled tracheal tube placement. General anesthesia was maintained with intravenous propofol and inhalational sevoflurane. Dissection of the excised larynx verified the distorted structure of the glottis with an S-shaped stenosis.

## Discussion

Securing airway is essential for the management of anesthesia in head and neck surgery, and reinforced evaluation of the airway must be performed preoperatively. For recurrent laryngeal cancer surgery, special considerations are necessary, and a safe airway management strategy should be planned in cases of stenosis, metastasis, contracture of the previous incision scar and dermal tension in the cervical area resulting from radiation therapy (8–11). In our three reported cases, only one of them was intubated successfully, while the other two patients had a very difficult airway anatomy that even limited the fibroscopy.

In the first case, the unanticipated scenario of can't intubate, can't ventilate (CICV) occurred, suggesting that routine airway assessment may have failed to detect the anatomic variation after partial laryngectomy and posed a potentially extreme risk for airway management of anesthesia (12). Deformation of the glottis in case one may have invalidated LMA ventilation and progressed into the urgent airway. Under such circumstance, pharmacologic intervention cannot be relied upon and rapid recovery of spontaneous ventilation was impossible to acquire. Thus, immediate surgical airway was demanded.

For all the patients before surgery, the examination of the laryngeal structures has been done under fibroptic laryngoscopy by an otolaryngologist, but was mainly focused on the local lesion. The tip of the fibroptic laryngoscope is adjacent to the lesion, but the global visualization of the pharyngeal and laryngeal structures might not be available. However, for the anesthesiologists, preoperative vision and detailed evaluation of the airway including the epiglottis and glottis, the existence and form of tumor, bleeding and edema is paramount. Therefore, fibroscopy before anesthesia, to examine the condition of larynx and pharynx, is indispensable regardless of the plan for normal induction of anesthesia or conscious fibroscopic assisted intubation (10, 13). In the 10-years period experience of Moorthy SS, fibroscopy was used for airway evaluation before anesthesia in every case in the management of airway in patients with laryngeal tumors (14).

Our point of view is that tracheal intubation after normal induction is possible if the glottis is well viewed under fibroscopy when other possibilities of difficult airway are excluded such as metastasis. If the patient has mild apnea, but the glottis can be viewed by fibroscopy, then fibroscopic tracheal intubation under adequate topical anesthesia and appropriate sedation following mask oxygen delivery is indicated. However, it was reported that in severe conditions, smaller changes of patient position might lead to complete airway obstruction and subsequent cardiac arrest (7). In addition, it is advisable that bleeding or scrapping of the tumor should be avoided to keep a clear view and prevent sudden suffocation during fibroscopy for airway evaluation or tracheal intubation. As an alternative way of airway evaluation, video laryngoscopy has a broader view, but may induce a more potent stimulus than fibroscopy. During active bleeding of supraglottic tumor, video laryngoscopy is suggested (15). On severe pharyngeal edema while difficult tracheal intubation was anticipated, it was reported that intubation through an air-Q intubating laryngeal airway successfully secured ventilation with preserved spontaneous breathing (16).

For some laryngeal cancer patients, supine apnea that resolves by lateral or prone positioning may indicate a pharyngeal, cervical or anterior mediastinal mass (17). Acute airway obstruction is a risk for patients in supine position when induction of anesthesia begins without a detailed assessment of airway risk. For laryngeal cancer patients with wheezing, airway evaluation by laryngoscopy and bronchoscopy is also requested.

In our report, the second case with inspirational apnea had a metastatic tumor located in the area of the tracheostomy with the concomitant invasion of the trachea. Thus conscious tracheostomy performed under local anesthesia could have aggravated his apnea and lead to a life-threatening scenario. According to the ASA practice guideline, fiberoptic-guided intubation was successful 87–100% of patients with difficult airway (5). Furthermore, fiberoptic-guided intubation under local anesthesia was described as an easy and safe technique to secure the airway in patients with upper airway tumors (18). Therefore, the risk for the airway may be more appropriately prevented by preoperative fibroptic-guided intubation when a difficult airway is anticipated and tracheostomy is contraindicated as our second case. In addition, according to the study of Toyota K, spiral CT scan is helpful to evaluate the degree of tumor-induced tracheal stenosis (19).

As reported by Fritzsche K, the relatively safer measure, when the supraglottic lesion blocks the view of glottis, is jet ventilation through the approach of tracheostomy, which offers an alternative to the endotracheal tube (20). In cases when severe pharyngeal edema is present, the subglottic tumor induces critical apnea, and marked three depression signs, but the anesthesiologist has a good vision of glottis, LMA placement followed by intra-tracheal stenting via the course of LMA is suggested to manage the difficult airway (21). Under these circumstances, if further surgery is still indicated, the application of either fibroscopic-guided intubation with proper small size tracheal tube after adequate oxygen delivery, or partial resection of the lesion under fibroscopy through the course of LMA are recommended (22). Moreover, to avoid malpractice, consultation with the surgeon prior to airway management is mandatory in clinical setting (13).

As part of the treatment strategy for laryngeal cancer, radiation therapy after the primary surgery may lead to immobility of the mandible and neck (23, 24). The clue of a difficult airway may be identified through careful examination of the area between the hyoid bone and submentum. This can be explained by the fact that the compliance of the submandibular structures may be decreased by radiation-induced fibrosis (25). For some patients, even with normal mentohyoid distance, the larynx might be located much more anterior than normal, due to radiotherapy-induced fibrosis in the submandibular area (26, 27). Moreover, the radiotherapy can also impair the lymphatic ducts and contribute to local edema while sometimes, acute side effects of radiation therapy result in dermatitis, oral mucositis which can make the tissue more susceptible to infection, and bleeding may occur during airway management (28, 29). Therefore, as the anatomy of the airway can be altered by the primary surgery and the corresponding radiation therapy, a difficult airway may present for the secondary surgery and preoperative tracheostomy sometimes becomes indicated. This safety issue was concerning in the third case, after the failed intubation attempt even with a slim fibroscope, due to glottic anatomic alteration caused by the lesion in the submandibular area. Therefore, preoperative tracheostomy with superficial cervical plexus block was suggested and mask oxygen supply was offered. However, tracheostomy may affect tumor growth, the range of dissection, and staging. Meanwhile, intraoperative tracheostomy is often an integral part of the surgery. By balancing the patient comfort and safe airway, superficial cervical plexus block, combined with topical intratracheal anesthesia under light sedation, seemed to be an appropriate option for the patient in the third case.

## Conclusion

Due to the deformation of the larynx following partial laryngectomy and invasion of the tumor as well as radiation-induced submandibular fibrosis, the recurrent laryngeal cancer may pose a risk of resulting CICV as in our first case which demands urgent surgical airway performance during induction of anesthesia for the secondary total laryngectomy. According to our second reported case, fiberoscopy may play an important role in evaluating and resolving such difficult airway management. If fiberoptic-guided tracheal intubation is anticipated to be difficult as in our third case, tracheostomy should be considered to secure the difficult airway.

## Ethics statement

Ethics approval from Ethic Committee of Wenzhou Medical University and consent to participate: Written patient consents were obtained for the publication of this case.

## Author contributions

XZ, OC, and YZ conducted a review of the literature. XZ, YZ, and SD prepared the body of the manuscript. XZ and OC critically reviewed the publication. All authors endorsed the final form of the manuscript.

### Conflict of interest statement

The authors declare that the research was conducted in the absence of any commercial or financial relationships that could be construed as a potential conflict of interest.

## Abbreviations

ASA: American society of anesthesiology
ECG: Electrocardiogram
SPO2: Saturation of pulse oxygen
BP: Blood pressure
LMA: Laryngeal mask airway
CICV, Can't intubation: can't ventilation
CT: Computed tomography.
